# Special Issue on Flavour Volatiles of Wine

**DOI:** 10.3390/foods11010069

**Published:** 2021-12-29

**Authors:** Matteo Bordiga, Fulvio Mattivi

**Affiliations:** 1 Department of Pharmaceutical Sciences, Università degli Studi del Piemonte Orientale “A. Avogadro”, Largo Donegani 2, 28100 Novara, Italy; 2 Department of Cellular, Computational and Integrative Biology (CIBIO), University of Trento, Via Sommarive 9, Povo, 38123 Trento, Italy; fulvio.mattivi@unitn.it; 3 Department of Food Quality and Nutrition, Research and Innovation Centre, Fondazione Edmund Mach, 38098 San Michele all’Adige, Italy

The perception of wine flavour and aroma is the result of several interactions between a large number of chemical compounds and sensory receptors. Compounds show synergistic (one compound enhances the perception of another) and antagonistic (one compound suppresses the perception of another) interactions. The chemical profile of a wine is derived from the entire process, starting from the grapes until bottled ageing. At the moment, wine makers are limited as to the range of yeasts that are able to impart some specific aromatic characteristic to a wine. Research focuses on issues such as adjusting the levels of flavour and aroma compounds, in particular esters and alcohols, producing enzymes that will release additional volatile compounds from the grapes, and reducing the amount of alcohol to levels that allow a better perception and release in the headspace of aroma and flavour compounds. New yeast strains are continuously being developed by traditional breeding techniques, leading to different flavour and aroma profiles in wine. In this context, the aim of the present Special Issue was to invite colleagues to submit their original research or review articles covering novel aspects of volatile compound research in the wine sector. Potential flavour volatiles of wine include: (i) varietal; (ii) pre-fermentative; (iii) formed by the yeast during alcoholic directly related to alcoholic fermentation; (iv) related to amino acid metabolism; (v) formed during malolactic fermentation; (vi) formed during ageing (reductive and oxidative pathway) and maturation. The aim was also to reach a mechanistic understanding of these pathways, with a focus on the reactions involved in the formation or degradation of key wine odorants and of the technological factors involved during the winemaking process.

As a result, the final SI results in a balanced collection of original scientific research papers covering a broad range. A study reported the aroma profiles of withered Corvina and Corvinone wines from two different Valpolicella terroirs in relationship to yeast strain and the use of spontaneous fermentation [1]. Interestingly, in this study, the spontaneous fermentation reduced the sensory properties associated with the grape origin and variety, possibly due to the overproduction of acetic acid and ethyl acetate. Another study evaluated the effect of microwave treatment in grape maceration (at a laboratory scale) on the content of free and glycosidically bound varietal compounds of must and wines and on the overall aroma of wines produced with and without SO_2_ [2]. In the study performed by Amores-Arrocha et al. (2021), a comparative study using bee pollen versus commercial fermentation activators in white and red winemaking evaluated promising new applications of this natural product [3]. 

Meanwhile, in order to differentiate white wines from Croatian indigenous varieties, the volatile aroma compounds were isolated by headspace solid-phase microextraction (HS-SPME) and analysed by comprehensive two-dimensional gas chromatography with time-of-flight mass spectrometry (GC×GC-TOF-MS) and conventional one-dimensional GC-MS [4]. More than 1000 compounds were detected and ca. 350 annotated. Several monoterpenes were proven particularly useful to differentiate among the wines. 

Another study, for example, reports noteworthy data on the composition and sensory profiles of white wines made from the novel grape genotypes Albillo Dorado and Montonera del Casar (*Vitis vinifera* L.) [5]. Interestingly, Abreu et al. (2021) realised a comprehensive review reporting the description of the most important technological, chemical and sensory characteristics of some of the main fortified wines: Madeira, Port, Sherry, Muscat, and Vermouth [6].

This SI, therefore, provides a state-of-the-art overview of novel aspects of volatile compound research in the wine sector. We hope it will attract the interest not only among researchers and winemakers but also among the students in viticulture and oenology anxious to cover results in the frontiers of research that are not yet covered in the textbooks. This SI could not have been realised without the inputs of our valued contributors, all experts in the field, and the editorial team. We thank them for their willingness to contribute. Likewise, we are most indebted for the critical reviews from our peer reviewers.
